# Organic-Inorganic Hybrid Materials for Room Temperature Light-Activated Sub-ppm NO Detection

**DOI:** 10.3390/nano10010070

**Published:** 2019-12-28

**Authors:** Abulkosim Nasriddinov, Marina Rumyantseva, Tatyana Shatalova, Sergey Tokarev, Polina Yaltseva, Olga Fedorova, Nikolay Khmelevsky, Alexander Gaskov

**Affiliations:** 1 Chemistry Department, Moscow State University, Moscow 119991, Russia; naf_1994@mail.ru (A.N.); shatalovatb@gmail.com (T.S.); pergeybokarev@gmail.com (S.T.); yal-polina@yandex.ru (P.Y.); fedorova@ineos.ac.ru (O.F.); gaskov@inorg.chem.msu.ru (A.G.); 2 Faculty of Materials Science, Moscow State University, Moscow 119991, Russia; 3 A.N. Nesmeyanov Institute of Organoelement Compounds RAS, Moscow 119991, Russia; 4 LISM, Moscow State Technological University Stankin, Moscow 127055, Russia; khmelevsky@mail.ru

**Keywords:** organic–inorganic hybrid materials, tin dioxide, indium oxide, Ru (II) complex, nitrogen monoxide NO, semiconductor gas sensor, room temperature, visible light activation

## Abstract

Nitric oxide (NO) is one of the main environmental pollutants and one of the biomarkers noninvasive diagnosis of respiratory diseases. Organic-inorganic hybrids based on heterocyclic Ru (II) complex and nanocrystalline semiconductor oxides SnO_2_ and In_2_O_3_ were studied as sensitive materials for NO detection at room temperature under periodic blue light (λ_max_ = 470 nm) illumination. The semiconductor matrixes were obtained by chemical precipitation with subsequent thermal annealing and characterized by XRD, Raman spectroscopy, and single-point BET methods. The heterocyclic Ru (II) complex was synthesized for the first time and characterized by ^1^H NMR, ^13^C NMR, MALDI-TOF mass spectrometry and elemental analysis. The HOMO and LUMO energies of the Ru (II) complex are calculated from cyclic voltammetry data. The thermal stability of hybrids was investigated by thermogravimetric analysis (TGA)-MS analysis. The optical properties of Ru (II) complex, nanocrystalline oxides and hybrids were studied by UV-Vis spectroscopy in transmission and diffuse reflectance modes. DRIFT spectroscopy was performed to investigate the interaction between NO and the surface of the synthesized materials. Sensor measurements demonstrate that hybrid materials are able to detect NO at room temperature in the concentration range of 0.25–4.0 ppm with the detection limit of 69–88 ppb.

## 1. Introduction

Nitric oxide (NO) and nitrogen dioxide (NO_2_) gases are among the main environmental pollutants produced from transports, industrial activities, and at high temperatures during the combustion processes [1]. Approximately 90% of nitrogen oxides are formed in the form of nitrogen monoxide, the remaining 10% is attributed to a strong oxidant and corrosive gas—nitrogen dioxide. However, a significant part of NO is oxidized in air in ambient conditions by oxygen, ozone, and VOCs to form NO_2_. The NO_x_ gases in indoor and outdoor air may cause respiratory and lung diseases [2,3], allergy and eczema [4], acid rain [5], photochemical smog [6].

The great interest in the quantification of nitric oxide NO is due to the fact that this molecule is one of the key regulators of cellular metabolism in the human body. On the other hand, exhaled NO is a biomarker of inflammatory respiratory and lung diseases, including asthma and chronic obstructive pulmonary disease (COPD). Analysis of NO concentration in exhaled air is a promising approach in noninvasive diagnosis of respiratory diseases [7,8,9]. In this case, the limitation of the quantitative determination of NO in exhaled air is due to the low level of its concentration (20–200 ppb) among a wide range of interfering gases [10].

Direct determination of nitrogen monoxide in the gas phase is possible using conductometric gas sensors based on various semiconductor metal oxides [7,10,11,12,13,14,15,16,17,18,19,20,21,22]. To increase selectivity of such analysis along with lower power consumption, complete or partial replacement of thermal heating with photoactivation is a promising approach. Activation of the sensor response under illumination occurs through various mechanisms depending on the nature of the target gas. For oxidizing gases (NO_2_, O_3_), which compete with oxygen for the same adsorption sites, photogeneration of electron-hole pairs plays a major role in the photodesorption process. On the contrary, for the detection of reducing gases (CO, NH_3_, H_2_S), the presence of chemisorbed oxygen on the surface of the semiconductor oxide is necessary. In this case, the increase in the sensor response under illumination is due to the unpinning of Fermi level of the semiconductor. In most works NO is detected as oxidizing gas. The similar routes of NO and NO_2_ sensing was recently evidenced by in situ DRIFT spectroscopy [23]. Our previous works show that highly selective NO_2_ detection is possible using visible light photoactivation [24,25,26,27]. To shift the optical sensitivity of semiconductor oxides into the visible range, it is necessary to create defects in the semiconductor matrix or introduce photosensitizers. The use of quantum dots-semiconductor nanocrystals, the optical properties of which depend on their size, allows to vary the light energy corresponding to the maximum absorption of the sensitizer. A similar effect is achieved when creating hybrid materials in which the photosensitizer is an organic dye. However, in this case, there is an additional advantage: in addition to the generation of photoexcited charge carriers and their spatial separation, the appearance of a specific activity of the organic component in reactions with gas phase molecules is possible.

Ru (II) heterocyclic complexes are among the most effective photosensitizers due to its electrochemical and photophysical properties, high molar extinction coefficient in the visible range, long lifetime of the excited state, and high luminescence intensity [28,29,30,31,32,33]. Comparison of the sensor properties of nanocomposites based on nanocrystalline oxides ZnO, SnO_2_, In_2_O_3_, and CdSe quantum dots in room temperature NO_2_ detection under visible light photoactivation showed that semiconductor matrices SnO_2_ and In_2_O_3_ provide higher sensitivity of nanocomposites toward NO_2_ [25]. In present work, the hybrid materials based on nanocrystalline SnO_2_ and In_2_O_3_ and Ru (II) heterocyclic complex as the photosensitizer were studied as sensitive materials for sub-ppm NO detection at room temperature under periodic illumination with blue light (λ_max_ = 470 nm) corresponding to the MLCT transition in photosensitizer.

## 2. Materials and Methods

### 2.1. Materials Synthesis

#### 2.1.1. Synthesis of Ru (II) Complex

The heteroleptic Ru (II) complex bis(2,2′-bipyridin-k^2^N^1^,N^1^′)[4-{(E)-2-[5-(1H-imidazo[4,5-f] [1,10]phenanthrolin-2-yl)thiophen-2-yl]ethynyl}-1-methylpyridinium iodide] ruthenium (II) dichloride (RuITP) was prepared by several steps as shown in Schemes S1–S3 (Supplementary Materials). The synthesized ligand and its respective heteroleptic ruthenium complex were characterized by ^1^H NMR, ^13^C NMR, MALDI-TOF mass spectrometry, and elemental analysis (Supplementary Materials).

#### 2.1.2. Synthesis of Nanocrystalline SnO_2_ and In_2_O_3_

Nanocrystalline SnO_2_ was synthesized by chemical precipitation method [25]. Tin (IV) chloride pentahydrate SnCl_4_·5H_2_O (15.00 g, 98%, Sigma-Aldrich, St. Louis, MO, USA) was dissolved in deionized water (150 mL). Aqueous ammonia (25%) was added dropwise to the resulting solution with vigorous stirring with a magnetic stirrer until pH = 7. The synthesis was carried out at room temperature. The gel-like precipitate was separated by centrifugation (3500 rpm, 3 min.), washed several times with deionized water and then with 0.01 M solution of NH_4_NO_3_ (99%, Sigma-Aldrich) until complete removal of chloride anions (AgNO_3_ test). The resulting precipitate of the α-stannic acid gel was dried at 100 °C for 24 h, then the vitreous product was ground in an agate mortar and annealed in air at 300 °C for 24 h.

To obtain nanocrystalline In_2_O_3_, aqueous ammonia solution (25%) was added dropwise to the stirred solution of In(NO_3_)_3_·4.5Н_2_O (5.00 g in 50 mL deionized water) until pH = 7 at room temperature. The resulting white gel of indium (III) hydroxide was separated by centrifugation and repeatedly washed several times with deionized water until the beginning of the peptization process. The obtained precipitate was dried at 100 °C for 24 h, then was ground in an agate mortar and annealed in air at 300 °C for 24 h.

#### 2.1.3. Synthesis of Hybrid Materials

Hybrid materials were formed by adsorption of Ru (II) on the surface of semiconductor oxides. This method provides, on the one hand, direct contact between the photosensitizer and the semiconductor matrix, and on the other hand, allows to keep open the part of the surface of the semiconductor oxide for interaction with the gas phase.

Hybrid materials based on nanocrystalline SnO_2_ and In_2_O_3_ and RuITP heterocyclic complex were prepared by two different procedures:

(i) in the form of powders to investigate optical properties and thermal stability. The RuITP organic dye was dissolved in methanol, and then 10 μL of the prepared solution was added dropwise to the weighed SnO_2_ or In_2_O_3_ powder. The obtained paste was dried at room temperature, the addition of each subsequent portion of the solution was carried out after complete evaporation of the solvent. The concentration of the solution was adjusted so that the Ru content in the hybrid material was 1 wt.%.
(ii) in the form of thick films over specially designed micro-hotplates, provided with Pt contact electrodes and Pt contact heater to investigate gas sensor properties. Weighed nanocrystalline SnO_2_ or In_2_O_3_ powder was mixed with a vehicle (α-terpineol in ethanol). The paste obtained was deposited by microdroping technique (GeSim multi-dos 2 with NanoTipHV head) on the surface of microhotplate to cover the contact electrodes. Thick films were dried at 50 °C for 24 h and sintered at 300 °C for 24 h in air. The thickness of the films, estimated from the preliminary calibration carried out by scanning electron microscopy, was about 1 μm. The obtained films were functionalized by RuITP methanol solution and dried at 50 °C for 24 h in air.

### 2.2. Materials Characterization

The phase composition of the nanocrystalline SnO_2_ and In_2_O_3_ was confirmed by X-ray powder diffraction (XRD) using DRON-4 diffractometer (Burevestnik, Moscow, Russia) with Cu K_α1_ radiation (λ = 1.54059 Å) and Raman spectroscopy using Renishaw InVia multichannel spectrometer (Renishaw plc., Wotton-under-Edge, UK) with an argon laser (λ = 514 nm) as the radiation source. The crystallite size (d_XRD_) of the SnO_2_ and In_2_O_3_ was calculated from the broadening of the most intense XRD peaks using Scherer equation. The specific surface area (S_surf_) and pore size distribution were determined by nitrogen adsorption with the ASAP 2020 instrument (Micromeritics Inc., Norcross, GA, USA). The surface area available for adsorption was calculated from the Brunauer–Emmett–Teller (BET) model. The pore size distribution was determined using Barrett–Joyner–Halenda (BJH) model.

The microstructure of the powders of semiconductor oxides was characterized by high resolution transmission electron microscopy (HRTEM). The study was performed at a Tecnai Osiris transmission electron microscope (FEI Company, Hillsboro, Oregon, USA) FEI Osiris microscope (FEI Company, Hillsboro, OR, USA) operated at 200 kV. The morphology and composition of thick films of hybrid materials were characterized by scanning electron microscopy (SEM) with energy dispersive X-ray spectroscopy (EDX) using Zeiss NVision 40 (Carl Zeiss NTS GmBH, Oberkochen, Germany) microscope.

X-ray photoelectron spectroscopy (XPS) analysis was carried out on the XPS system (Thermo Fisher Scientific, Waltham, MA, USA) equipped with a hemispherical analyzer and using monochromatic Al Kα radiation as X-ray source (1486.7 eV).

UV/Vis absorption spectra of the Ru-ITP heterocyclic complex solution and hybrid materials’ powders were recorded in the wavelength range of 200–800 nm on Varian Cary 50 (Varian Inc., Melbourne, Australia) and Lambda-950 (Perkin Elmer Inc., Waltham, MA, USA), respectively. For liquid samples 10-mm quartz cell was used. Absorption spectra of the powders were measured using diffuse reflectance method. WS-1-SL Spectralon material (Labsphere, North Sutton, NH, USA) was used as diffuse reflectance standard for calibration. Thermogravimetric analysis combined with mass spectral analysis of gaseous products (TG-MS) was used to study thermal stability of the hybrid materials by STA 409 PC Luxx thermal analyzer with a quadrupole mass spectrometer QMS 403 C Aëolos (Netzsch-Gerätebau GmbH, Selb, Germany). The powders of hybrids based on semiconductor oxides and RuITP complex were heated up to 500 °C with the heating rate of 10 °C/min in air.

Diffuse reflectance infrared Fourier transform spectroscopy (DRIFTS) was performed to investigate the interaction between NO and the surface of the synthesized materials. DRIFTS measurements were performed on Perkin-Elmer Spectrum One Fourier Transform Infrared spectrometer (Perkin Elmer Inc., Beaconsfield, UK) with the DiffusIR annex and flow chamber HC900 (Pike Technologies, Cottonwood Dr., Madison, WI, USA) in the range of 4000–400 cm^−1^ with a resolution of 4 cm^−1^ and accumulation of 30 scans. The window of chamber was made of KBr disc (32 mm diameter, 3 mm thickness). Samples (30 mg) were placed in ceramic crucibles (5.0 mm diameter, 2.0 mm depth), set into the heat chamber attached with water coolant line, preheated to 50 °C for 1 h and then cooled down to the room temperature. The DRIFT spectra were recorded at room temperature under a controlled gas flow rate of 100 mL/min. The gas mixture containing 50 ppm of NO was prepared by dilution of certified gas mixture 100 ± 5 ppm of NO in N_2_ (Monitoring, St. Petersburg, Russia) with background purified air from a pure air generator (Granat-Engineering Co. Ltd., Moscow, Russia).

Electrochemical characterization of heterocyclic Ru (II) complex was effectuated at 22 °C using IPC-Pro M potentiostat (Volta, St. Petersburg, Russia) (Supplementary Materials). Cyclic voltammetry measurements (CVA) were carried out in a 1.0 mL cell equipped with a glassy carbon (GC) electrode (2.0 mm disk), Ag/AgCl/KCl (aq. saturated; reference electrode), and platinum electrode (counter electrode). The complex was dissolved in degassed dry CH_3_CN containing 0.1 M *tetra*-(butyl) ammonium perchlorate (Bu_4_NClO_4_) as the supporting electrolyte. Before cyclic voltammetry experiments dry argon gas was bubbled through the solutions for 30 min. The scan rate was 200 mVs^−1^. The calibration was performed using 10^−3^ M solution of ferrocene with 0.1 M TBAP in the same solvent.

The energies of HOMO and LUMO of RuITP complex were calculated using the methodology described in [34] by the following equations:

Е_HOMO_ = −4.73 − Е_onset(Ox)_ (1)

Е_LUMO_ = −4.73 − Е_onset(Red)_ (2)

Gas sensor measurements have been carried out at room temperature under constant flux of 100 mL/min controlled by electronic mass-flow controllers EL-FLOW F-221M (Bronkhorst, Netherlands). DC measurements using electronic module (MGA-2-1, Senseria, Russia) have been carried out in situ to monitor the electrical conductivity of the samples during exposure to NO/air (0.25–4.0 ppm NO in dry air) gas mixtures under periodic blue (λ_max_ = 470 nm) light illumination. Miniature LED of 20 mW/cm^2^ power (Star, Betlux Electronics Co., Ltd., Zhenhai, Ningbo, China) was used as an illumination source. An automatic cyclic relay (REV-114, Novatech-Electro, St. Petersburg, Russia) was used to open and close the circuit with period of 2 min, allowing illumination to be made in a pulsed mode. A detailed description of the specially designed micro-hotplates and sensor measurements setup is given in our previous work [27].

## 3. Results and Discussion

### 3.1. Characteristics of Nanocrystalline Semiconductor Oxides

According to the XRD data (Figure S5, Supplementary Materials), the investigated nanocrystalline metal oxides powders are single phase. The XRD patterns of the SnO_2_ corresponds to the tetragonal cassiterite structure (ICDD 41-1445), and In_2_O_3_ exhibits a cubic bixbyite structure (ICDD 6-416).

The N_2_ adsorption-desorption curves for nanocrystalline In_2_O_3_ and SnO_2_ (Figure S6, Supplementary Materials) can be attributed to type IV. In both cases the hysteresis is observed, that indicates the irreversible capillary condensation. According to the IUPAC classification, in the case of SnO_2_, the hysteresis is of the H1 type, which is characteristic for porous, spatially ordered structure that has minimal connectivity between adjacent pores. The hysteresis of the N_2_ adsorption-desorption curve of the nanocrystalline In_2_O_3_ is of the H2a type indicating that the pore cavity size distribution is wide compared with the neck size distribution [35]. For In_2_O_3_, the average pore size determined by the BJH model is 3–4 nm. In the case of SnO_2_ on the pore size distribution (Figure S7, Supplementary Materials) two maxima can be distinguished, corresponding to 3–5 nm and 70–80 nm. Comparison of particle size and pore diameter suggests that in the case of In_2_O_3_, the main contribution to the total volume is made by pores formed by crystallites, while for SnO_2_, the maximum volume is accounted for large pores formed by agglomerates.

**Figure 1:**
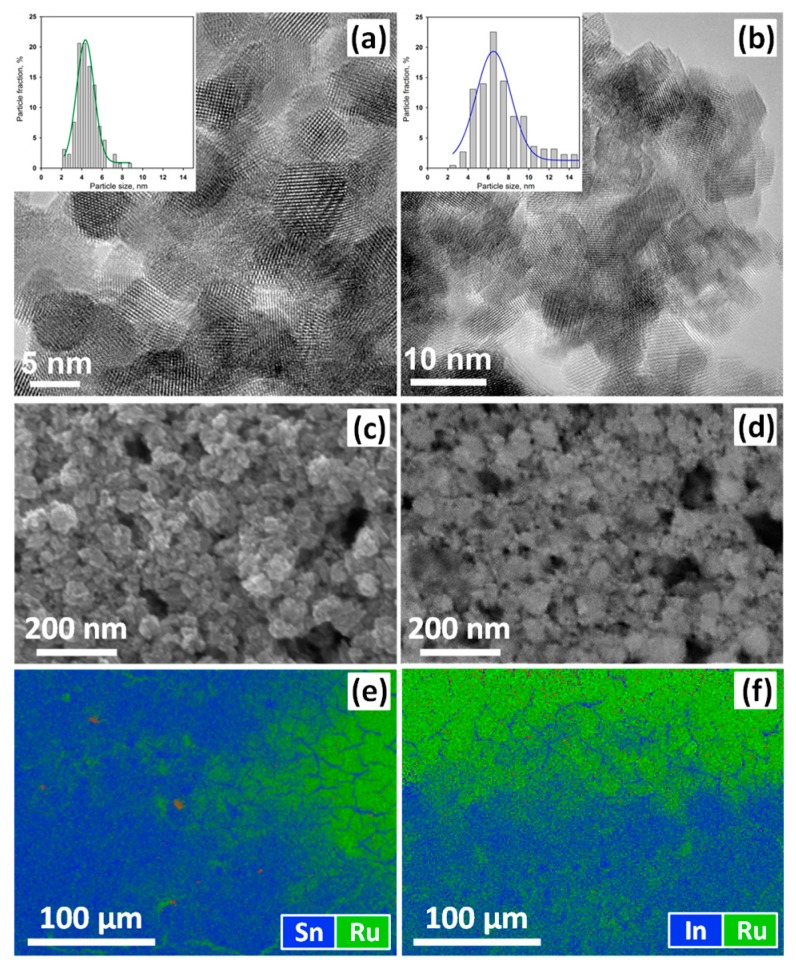
High resolution transmission electron microscopy (HRTEM) (**a**,**b**) images of SnO_2_ (**a**) and In_2_O_3_ (**b**) nanoparticles. SEM (**c**,**d**) images of SnO_2_ (**c**) and In_2_O_3_ (**d**) thick films deposited over functional substrates and sintered at 300 °C. Energy dispersive X-ray spectroscopy (EDX) maps (**e**,**f**) of element distribution on the surface of the hybrid SnO_2_+RuITP (**e**) and In_2_O_3_+RuITP (**f**) thick films.

HRTEM images and particle size distribution for the nanocrystalline oxides SnO_2_ and In_2_O_3_ are shown in Figure 1a,b. The obtained average particle sizes are in good agreement with the average size of the crystallites estimated by the Scherrer formula (Table 1). Figure 1c,d presents the micrographs of SnO_2_ and In_2_O_3_ thick films formed on the dielectric substrate of the measuring chip. Thick films are porous and consist of agglomerated and sintered grains. The size of agglomerates is about 100 nm. The microstructure characteristics of synthesized semiconductor oxides are presented in Table 1.

**Figure 2:**
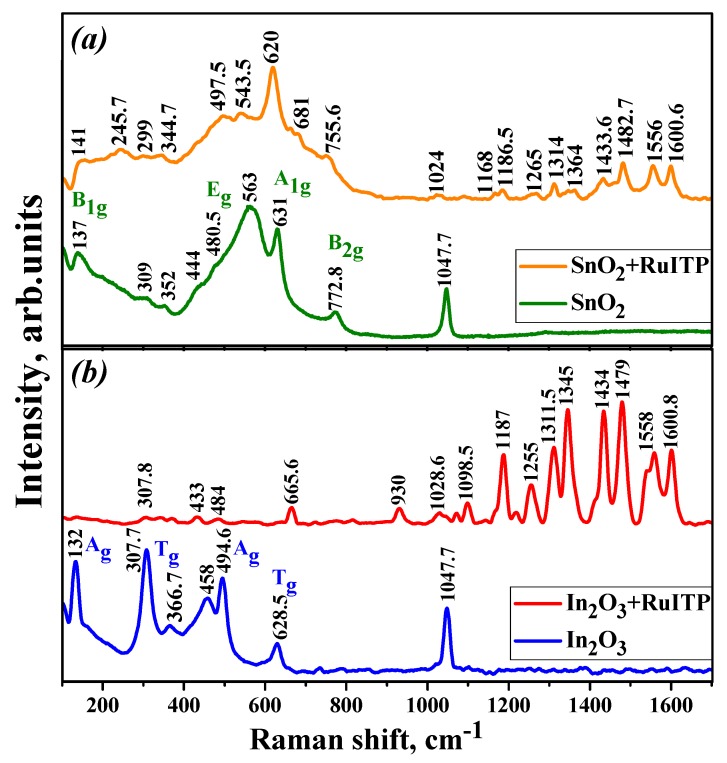
Raman spectra of nanocrystalline SnO_2_ (**a**) and In_2_O_3_ (**b**) based samples.

Figure 2a,b shows representative Raman spectra of the nanocrystalline SnO_2_ and In_2_O_3_ in the frequency range of 110–850 cm^−1^. Three characteristic vibrational modes of the tetragonal rutile structure of SnO_2_ are observed at 480.5 (E_g_), 631 (A_1g_), and 772.8 cm^−1^ (B_2g_) frequencies. The A_1g_ and B_2g_ modes are attributed to symmetric and asymmetric Sn–O stretching, respectively. The translational E_g_ mode is due to the oxygen anions motion along the *c*-axis [36,37]. The band at 137 cm^−1^ is associated with B_1g_ mode and appears only in the spectra of nanocrystalline SnO_2_ in the range of 100–184 cm^−1^ [38,39]. The second wide band at 563 cm^−1^ is associated with in-plane oxygen vacancies of the nanocrystalline SnO_2_ [40,41,42]. The bands at 309 and 352 cm^−1^ and the band at 444 cm^−1^ are assigned to the E_u_ and B_1u_ modes, respectively, associated with transformation of an IR to Raman active modes [43].

Characteristic Raman vibrational modes corresponding to the body-centered cubic In_2_O_3_ are observed at 132, 307.7, 366.7, 494.6, and 628.5 cm^−1^ and their positions are in agreement with previously reported data [44,45,46]. The peak at 132 cm^−1^ is attributed to the In-O vibration of [InO_6_] structural units, the peak at 307.7 cm^−1^ is due to the bending vibration of the δ-[InO_6_] octahedrons, the peaks at 494.6 and 628.5 cm^−1^ are assigned to the stretching vibrations of the ν-[InO_6_] octahedrons, and the peak at 366.7 cm^−1^ is attributed to the stretching vibrations of the In-O-In bonds [47,48,49]. The broad peak at 458 cm^−1^ probably has the same nature as SnO_2_ wide band at 563 cm^−1^ corresponding to a local structural defects due to small particle size of nanocrystalline In_2_O_3_ [50].

### 3.2. Characteristics of Ru (II) Heterocyclic Complex

**Figure 3:**
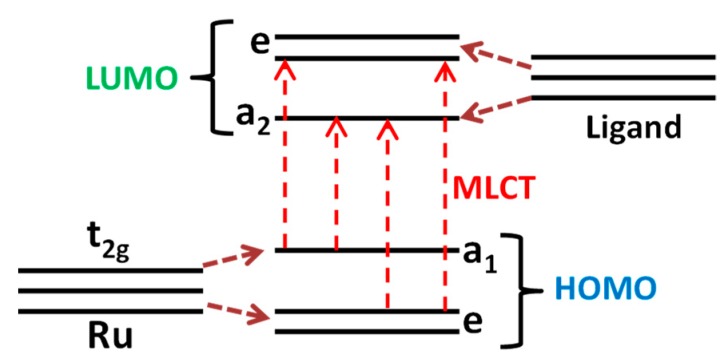
Energy level scheme of the molecular orbitals of Ru (II) heterocyclic complex. The four possible metal-to-ligand charge transfer (MLCT) states are indicated by the vertical arrows.

As a result of overlapping ruthenium d-orbitals with π*—anti-bonding ligand orbitals, the highest occupied molecular orbitals (HOMO) and the lowest unoccupied molecular orbitals (LUMO) are formed, as shown in Figure 3. As a result of the large energy difference between the occupied Ru d-orbitals and the unoccupied ligand orbitals, the HOMO is mainly localized at the metal and the LUMO belongs to the ligand anti-bonding orbitals. Therefore, the excitation and transition of an electron from the HOMO to the LUMO is called metal-to-ligand charge transfer (MLCT) and leads to the appearance of a photoabsorption band in the wavelength range between 380 and 520 nm [33,51]. MLCT transition corresponds to the visible spectral range; therefore, it is the most important characteristic of the complex molecule that can be used as a photosensitizer.

**Figure 4:**
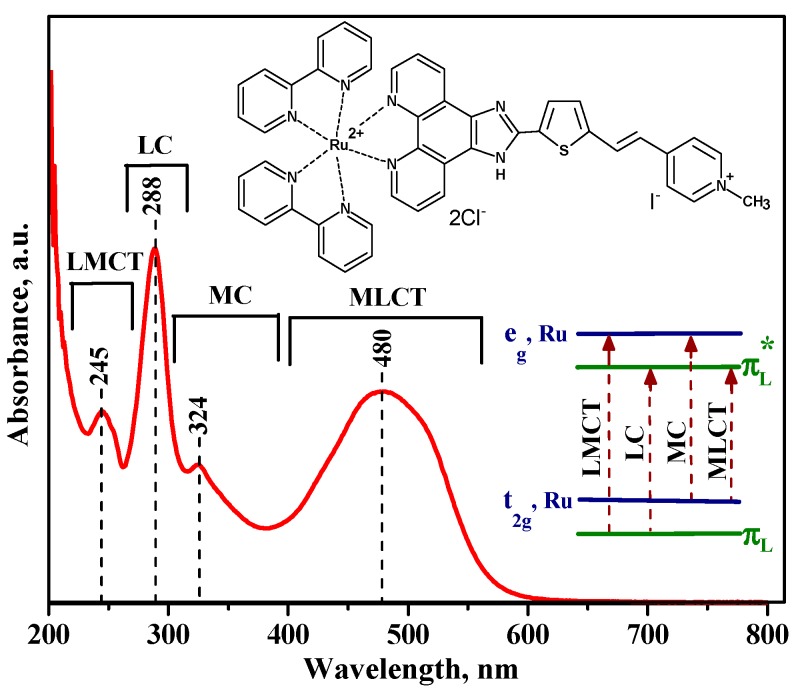
UV-Vis absorption spectra of the Ru (II) heterocyclic complex in methanol solution (C = 5 × 10^−5^ М). The inset shows the four possible transitions, labeled according to the involved molecular orbitals.

The optical absorption spectrum of Ru (II) heterocyclic complex is presented in Figure 4. The electronic transitions in this compound are observed both in the visible and in the near UV region. In addition to the MLCT transition, other important electronic transitions are summarized in the inset of Figure 4. The wide absorption band at 480 nm can be the result of overlapping of π → π* charge transfer in 1H-imidazo-phenantroline ligand and two MLCT processes: (t_2g_, Ru → π* substituted 1*H*-imidazo-phenantroline (ImPh) ligand) and (t_2g_, Ru → π* bpy ligand) transitions. In the UV range, high-energy transitions dominate in the process of intraligand π → π* charge transfer in bipyridine ligands (LC-ligand-centered) and ligand-to-metal charge transfer (LMCT) (π_L_ → e_g_, Ru). The weak absorption band at 324 nm can be attributed to the transition between the metal orbitals (MC-metal-centered) (t_2g_, Ru → e_g_, Ru) [33,52,53].

### 3.3. Characteristics of Hybrid Samples

The distribution of the Ru complex over the surface of SnO_2_ and In_2_O_3_ films was studied by EDX mapping (Figure 1e,f). For both hybrid materials, extended regions with uniform distribution of the photosensitizer over the surface of the semiconductor oxide were found.

**Figure 5:**
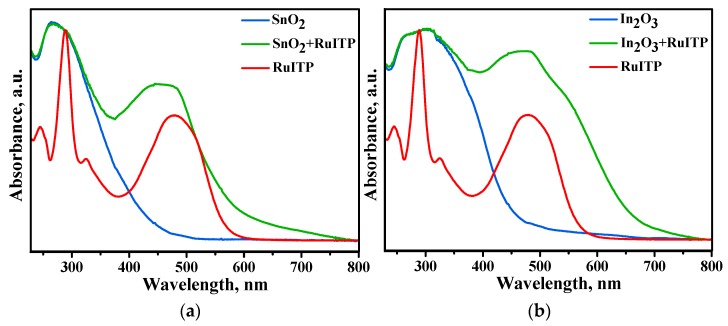
Absorption spectra of the RuITP dye, unmodified and sensitized nanocrystalline SnO_2_ (**a**) and In_2_O_3_ (**b**).

The optical properties of unmodified and sensitized nanocrystalline SnO_2_ and In_2_O_3_ were studied by UV-VIS spectroscopy (Figure 5). The results show that pure nanocrystalline SnO_2_ and In_2_O_3_ have an excellent optical transparency in the visible region. The strong absorption in the UV region between 260–360 nm wavelengths is attributed to the fundamental band gap electron transitions. In the spectra of hybrid samples the broad absorption band ranging from 370 nm to 600 nm is associated with RuITP complex. Compared with the spectrum of RuITP complex, a shift of ~20 nm to the short-wavelength region (blue shift) is observed, which may be due to the decrease in electron density on the Ru centers for hybrid materials. In addition, for In_2_O_3_ + RuITP sample a broad shoulder appears in the green region.

In the Raman spectra of hybrid materials (Figure 2), serial bands in the high-frequency region due to the Ru (II) heterocyclic complex were observed. The low-frequency region also consists of the contribution from SnO_2_ and In_2_O_3_ nanoparticles. The band at 1047.7 cm^−1^ in pure SnO_2_ and In_2_O_3_ probably attributed to longitudinal optical (LO) phonon vibrations. The wavelength of the laser used as the radiation source (514 nm) coincides with the wide shoulder of the In_2_O_3_+RuITP sample (Figure 5). This can lead to the resonance of the molecules in the excited state. Therefore, the bands associated with Ru (II) heterocyclic complex for In_2_O_3_+RuITP are more intense than for SnO_2_+RuITP.

The bands near 1600 cm^−1^ are assigned to valence vibrations of the C=C bonds of the bpy ligand rings mixed with the C-C links. The region between 1563–1540 cm^−1^ belongs to the vibration of the C=C and C=N bonds of the bpy cycles. The bands at about 1480 cm^−1^ are due to the C-C links vibrations that play a significant role in the electron transfer during light absorption [54,55]. The bands at 1310–1423 cm^−1^ associated with C-C inter-rings stretching vibration mixed with the deformation of the СН bonds [56]. There are a number of mixed vibrations in the middle region of the spectra. The C=C and C=N stretching frequencies are sensitive to the charge density on the bpy ligand [57]. Therefore, the spectral shifts in the low-frequency region can be associated with the interaction of the semiconductors with ruthenium (II) complex and the change in charge density.

Table 2 summarizes the observed frequencies of the Raman spectra for the nanocrystalline SnO_2_, In_2_O_3_, and hybrid materials according to [55,56,57].

**Figure 6:**
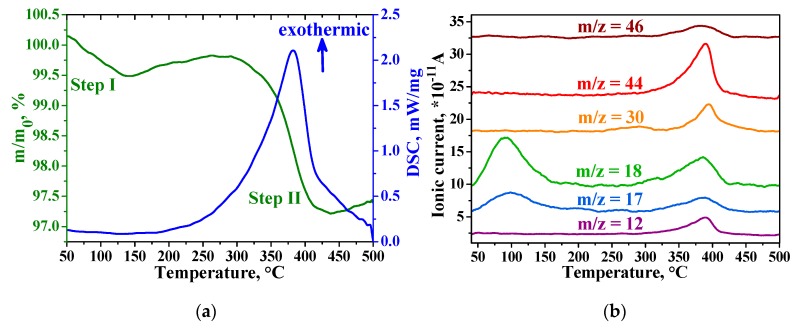
Thermal analysis (**a**) and mass spectrometry curves (**b**) of the In_2_O_3_+RuITP sample.

Thermogravimetric analysis (TGA) and mass spectrometry (MS) measurements data for In_2_O_3_+RuITP sample are shown in Figure 6. According to TGA, the thermal decomposition process of the sample proceeds in two steps (Figure 6a, green curve). The first decomposition step (Step I) is observed at the range of 35–150 °C, with a weight loss of 0.5%. The second step (Step II) with a weight loss of 2.8% occurs at 290–440 °C. Comparing with mass-spectra signals (Figure 6b) of the CO_2_ (*m*/*z* = 12, *m*/*z* = 44), H_2_O (*m*/*z* = 17, *m*/*z* = 18), NO (*m*/*z* = 30), NO_2_ (*m*/*z* = 46) one can conclude that “Step I” corresponds to the release of physically adsorbed water. “Step II” corresponds to the full exothermic decomposition of the Ru-ITP complex (Figure 6a, blue curve) over 200 °C which led to removal of CO_2_, H_2_O, NO, and NO_2_ from the hybrid material. TGA and MS data for the SnO_2_+RuITP sample have the same character.

### 3.4. Gas Sensor Properties

Based on previous studies, the following basic points describing the sensor properties of semiconductor oxides to NO and NO_2_ can be formulated:

(i) The interaction of *n*-type semiconductor oxides with NO_2_ is accompanied by a decrease in electrical conductivity, since nitrogen dioxide is an electron acceptor. It was shown by Raman spectroscopy [58] that the conductivity of *n*-type metal oxides in the presence of NO_2_ correlates with the concentration of surface bidentate nitrite groups. Surface groups observed on DRIFT spectra [23] suggest that NO_2_ adsorption on *n*-type semiconductor oxides occurs in the following possible ways:
  NO_2(gas)_ = NO_2(ads)_, (3)
  NO_2(gas)_ + *e*^−^ = NO_2_^−^_(ads)_, (4)
  2 NO_2(gas)_ + ½ O_2(gas)_ + 2 *e*^−^ = NO_2_^−^_(ads)_ + NO_3_^−^_(ads)_, (5)
  NO_2_^−^_(ads)_ + ½ O_2(gas)_ = NO_3_^−^_(ads)_. (6)
(ii) At room temperature under dark conditions, the adsorption of NO_2_ on the surface of *n*-type semiconductor oxides is an irreversible process: when NO_2_ is removed from the atmosphere, there is no return of the conductivity (resistance) to its initial value in pure air [24,59]. The role of illumination probably consists in the photogeneration of holes that ensure the conversion of chemisorbed NO_2_^−^ particles into physically adsorbed NO_2_, which can be desorbed from the surface of the semiconductor oxide even at room temperature.
(iii) Usually, NO is also detected by various n-type semiconductor metal oxides as an oxidizing gas. Based on the results obtained by DRIFT method, the authors [23] suggested that NO sensing by semiconductor oxides is determined by the following reaction:
  NO_(gas)_ + ½ O_2(gas)_ + *e*^−^ = NO_2_^−^_(ads)_. (7)

**Figure 7:**
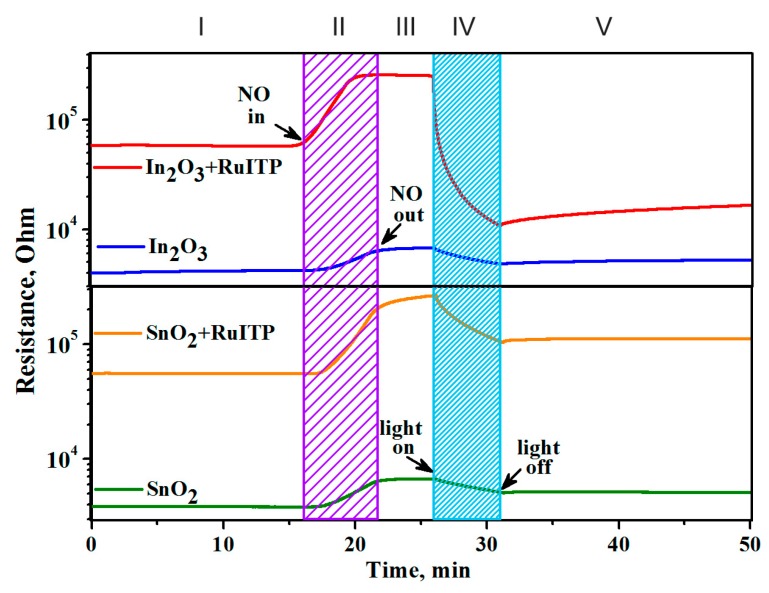
Room temperature resistance of unmodified oxides SnO_2_ and In_2_O_3_ and hybrids SnO_2_+RuITP and In_2_O_3_+RuITP in the presence of 4 ppm NO in dark conditions and under blue light LED illumination (λ_max_ = 470 nm). I—air, dark conditions; II—in the presence of target gas, dark conditions; III—air, dark conditions; IV—air, light illumination; V—air, dark conditions.

Figure 7 reveals the effect of blue light (λ_max_ = 470 nm) on the interaction of semiconductor oxides SnO_2_ and In_2_O_3_ and hybrid materials with NO at room temperature. In dark conditions in pure air the resistance of samples is constant. When the target gas (4 ppm NO) is introduced into the atmosphere (Figure 7, II), the resistance of all samples increases due to reaction (7). The reached resistance values remain nearly constant when NO is removed from the measurement cell (Figure 7, III). So, at room temperature in dark conditions, adsorption of NO on the surface of semiconductor oxides proceeds irreversibly similarly to NO_2_ [24].

Blue light illumination (Figure 7, IV) results in the decrease in the resistance for all samples. This indicates elimination of NO_2_^−^ from the surface of semiconductors:

NO_2_^−^_(ads)_ + *h*^+^ = NO_2(gas)_. (8)

This effect is more pronounced for hybrid SnO_2_+RuITP and In_2_O_3_+RuITP samples as compared with corresponding pure oxides SnO_2_ and In_2_O_3_. For In_2_O_3_+RuITP sample the achieved resistance value is even lower than that in dark conditions. For this sample, turning light off (Figure 7, V) leads to the very slow resistance increase tending to the dark value. This additional change in the resistance probably is due to chemosorption of oxygen in dark conditions (Equation (9)) and its photodesorption under illumination (Equation (10))

O_2(gas)_ + *e*^−^ = O_2_^−^_(ads)_, (9)

O_2_^−^_(ads)_ + *h*^+^ = O_2(gas)_. (10)

Note that for pure oxides SnO_2_ and In_2_O_3_ there is a decrease in resistance under blue light. The photosensitivity of these materials to light with an energy (*E* = 2.63 eV) lower than the band gap (*E_g_*(SnO_2_) = 3.6 eV, *E_g_*(In_2_O_3_) = 3.5 eV) is obviously due to the presence of a large number of defects in nanocrystalline materials. This effect was considered in detail in our previous work [27]. Thus, when nitrogen monoxide NO is detected at room temperature, the use of illumination ensures that the electrophysical characteristics of hybrid materials are restored when the target gas is removed from the atmosphere.

**Figure 8:**
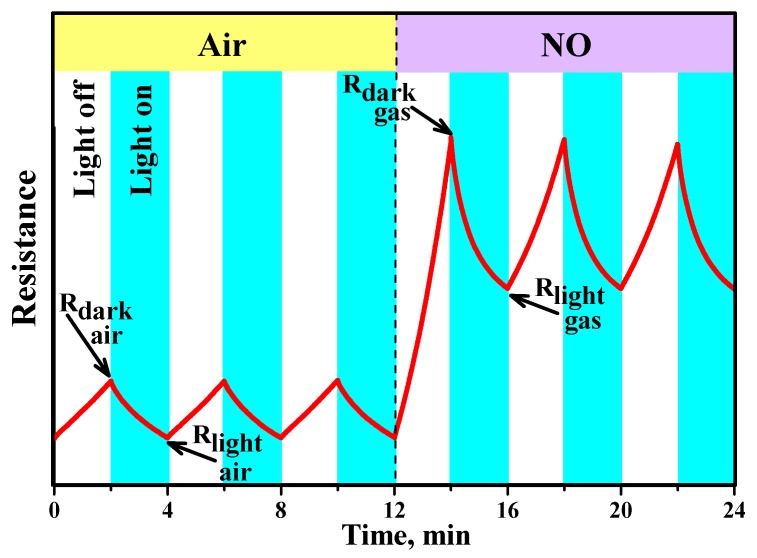
Scheme of changes in the resistance of semiconductor materials under periodic blue light LED illumination (λ_max_ = 470 nm) in pure air and in the presence of NO.

The sensor properties of the obtained nanocrystalline SnO_2_ and In_2_O_3_ and hybrid materials were studied in the concentration range of 0.25–4.0 ppm NO in dry air. The measurements were effectuated first with an increase and then a decrease in the concentration of the target gas. Gas sensor measurements were carried out under periodic illumination with periods of 2 min switching on and 2 min switching off the LED. Such periodic illumination results in the equivalent change in the sensors resistance, which becomes an alternation of the rise and fall curves of photoconductivity [27]. In the stationary state these curves are characterized by the minimum *R_light_* and maximum *R_dark_* resistances achieved when the sensor is illuminated and in dark conditions, respectively. This effective photoresponse *S_ph_* = *R_dark_*/*R_light_* depends on the target gas concentration and can be used as the sensor signal (Figure 8). The resistive response can be calculated [24] as the ratio *S* = *R_dark gas_*/*R_dark air_* of dark resistances (measured at the end of 2 min “light off” period, Figure 8) at a given NO concentration *R_dark gas_* and in pure air *R_dark air_*. This method of sensor signal calculation corresponds to maximum NO adsorption in dark conditions.

**Figure 9:**
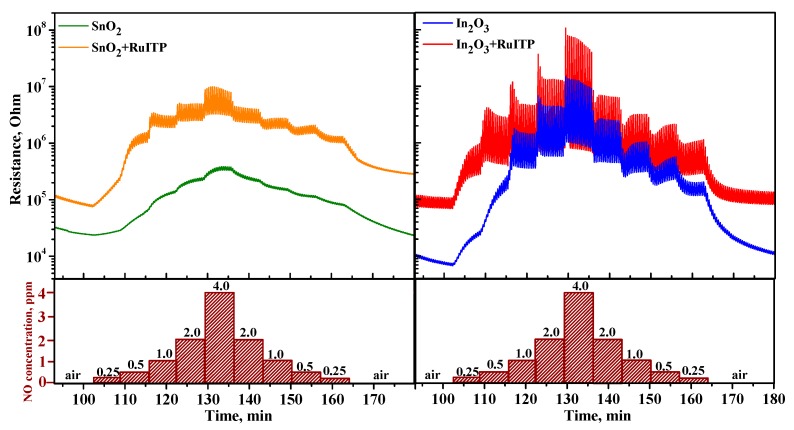
Room-temperature electrical resistance of the nanocrystalline SnO_2_ and In_2_O_3_ and hybrid samples under periodic illumination during stepwise change of the NO concentration.

Figure 9 presents the change of room temperature electrical resistances of nanocrystalline oxides SnO_2_ and In_2_O_3_ and hybrid samples SnO_2_+RuITP and In_2_O_3_+RuITP under periodic blue LED illumination during stepwise change of NO concentration. All samples exhibit similar behavior. Under illumination the resistance of the sensors decreases due to the discharge of chemisorbed anions by photogenerated holes following by desorption of adsorbed species. When the diode is switched off, the resistance of the sensors increases due to the chemisorption of oxygen and target gas accompanied by the capture of electrons from the conduction band of the semiconductor [25,26].

**Figure 10:**
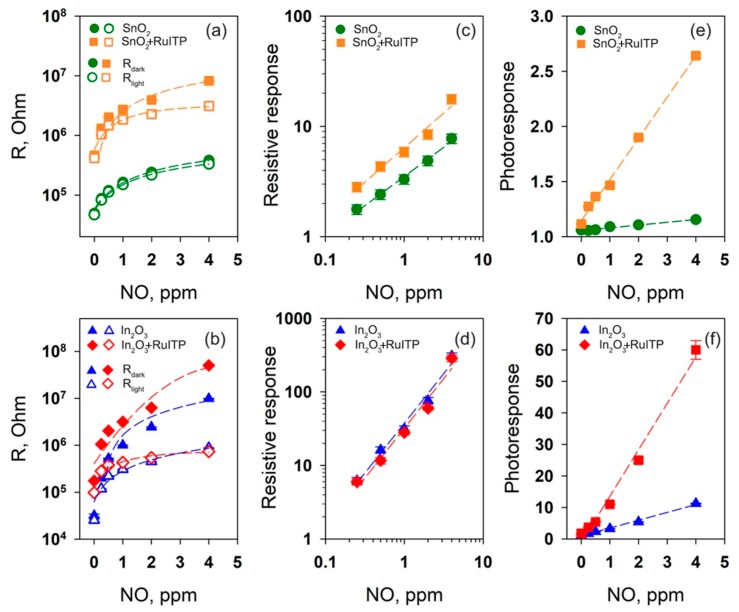
Room-temperature R_dark_ and R_light_ resistance values (**a**,**b**), resistive response (**c**,**d**), and photoresponse (**e**,**f**) depending on NO concentration for SnO_2_ based (**a**,**c**,**e**) and In_2_O_3_ based (**b**,**d**,**f**) samples.

The analysis of the data obtained is presented in Figure 10 and discussed below.

### 3.5. Influence of RuITP Complex on SnO_2_ and In_2_O_3_ Dark Resistance in Pure Air

**Figure 11:**
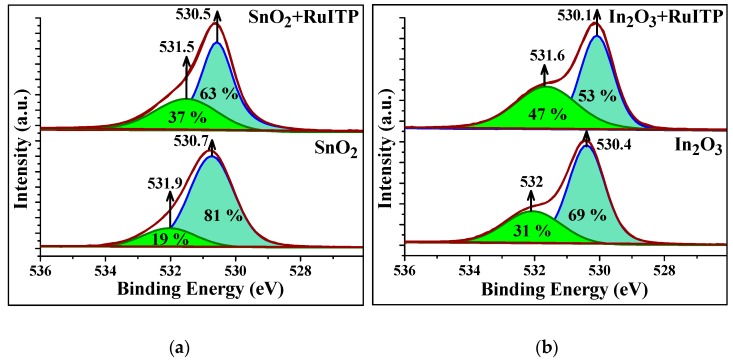
X-ray photoelectron spectra O 1s of the (**a**) SnO_2_-based and (**b**) In_2_O_3_-based samples.

First of all, it should be noted that modification of SnO_2_ and In_2_O_3_ by the RuITP complex leads to a significant (approximately 10-fold) increase in resistance under dark conditions in pure air (Figure 10a,b). To explain this effect, the surface of semiconductor oxides and hybrid materials was additionally investigated by XPS. The oxygen XP-spectra O 1s are shown in Figure 11. In all cases, the O 1s spectra can be deconvoluted into two components. The O(1) component (~530.5 eV) corresponds to the oxygen anions in the crystal structure of SnO_2_ and In_2_O_3_. The O(2) component (531.5–532.0 eV) is caused by the presence of various oxygen-containing species on the surface of materials. Modification of SnO_2_ and In_2_O_3_ with RuITP leads to an increase in the contribution of the higher-energy component O(2) from 19% to 37% in SnO_2_ based hybrids and from 31% to 47% in In_2_O_3_ based hybrids, indicating an increase in the concentration of chemisorbed oxygen on the surface of hybrid materials. Thus, the chemisorption of oxygen with electron capture of the conduction band of the semiconductor oxide (Equation (9)) leads to an increase in the resistance of hybrid samples compared to unmodified oxides SnO_2_ and In_2_O_3_.

### 3.6. Resistive Response in Dark Conditions

For all samples, the dependence of the resistive response on the NO concentration is linearized in double log coordinates (Figure 10c,d) that indicates their power-law nature, typical for the resistive type sensors $S\propto p_{NO_{2}}^{m}$. Using the average values (*R_dark av_*) and the standard deviation (σ) of the dark resistance in air from Figure 9 the detection limit of NO (LDL_NO_) has been determined from obtained calibration curves for each sensor. The value (*R_dark av_* + 3σ)/*R_dark av_* was taken as the minimum measurable resistive response. The LDL_NO_ values for SnO_2_, SnO_2_+RuITP, In_2_O_3_, and In_2_O_3_+RuITP samples were 140 ppb, 69 ppb, 91 ppb, and 88 ppb, respectively. Thus, modification of semiconductor oxides by the RuITP complex differently affects the sensitivity of SnO_2_ and In_2_O_3_ to NO under dark conditions.

**Figure 12:**
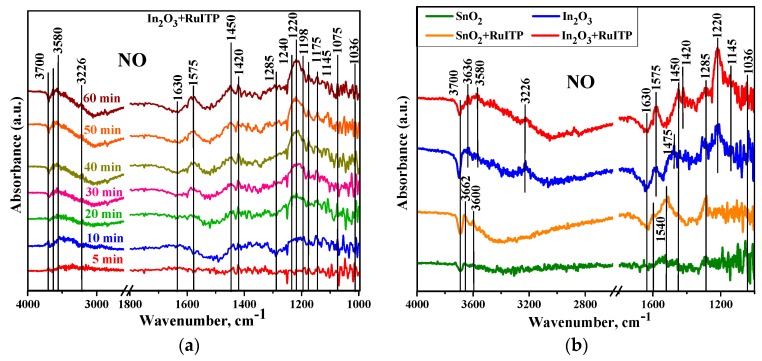
(**a**) In situ diffuse reflectance infrared Fourier transform spectroscopy (DRIFT) spectra of the In_2_O_3_+RuITP sample during NO (50 ppm) exposure at room temperature. (**b**) In situ DRIFT spectra of nanocrystalline SnO_2_ and In_2_O_3_ and hybrid materials after 60 min NO (50 ppm) exposure at room temperature.

To reveal the influence of RuITP complex on the interaction of semiconductor oxides SnO_2_ and In_2_O_3_ with NO, in situ studies were performed by DRIFTS method. Figure 12a depicts the time evolution of in situ DRIFT spectra at room temperature for In_2_O_3_+RuITP sample during NO (50 ppm) exposure. Firstly, during the adsorption process, the adsorbed species accumulate on free sites; almost all bands increased until saturation and then became stable. Some additional peaks were observed in the spectra of the In_2_O_3_+RuITP sample because of the more charge carriers (electrons) in the conduction band compared to other samples which leads to greater interaction with the typical Lewis acids. The peaks at 1145–1175 cm^−1^ and 1220–1240 cm^−1^ increased as well, indicating there is an accumulation of nitrite species and could be ascribed to corresponding vibrational modes of chelating and bridging bidentate NO_2_^−^ species [60].

The ν (N=O) vibrations of monodentate NO_2_^−^ species coordinated by its oxygen atoms can be assumed at the range of 1450–1475 cm^−1^ while coordinated via its N atom nitro compound NO_2_^−^ was detected at the frequency of 1420 cm^−1^. The monodentate NO_3_^−^ species result in absorption bands at the ranges of 1036–1050 cm^−1^, 1240–1285 cm^−1^, and 1540 cm^−1^. Absorption bands related to the chelating bidentate NO_3_^−^ anions are located in the region between 1575 and 1592 cm^−1^ [61]. At low temperatures, in particular at room temperature, the NO molecules tend to form dimers during adsorption [62]. Also in the presence of oxygen the molecule of nitrogen dioxide can be easily formed from nitrogen monoxide. However, in NO adsorption spectra (Figure 12b) there was not found any bands attributed to NO dimers and to nitrosyl anion (NO^−^) since it is difficult for NO molecule to accept an electron because of the existence of one unpaired electron on antibonding 2π orbital.

Since the preheating procedure was performed at 50 °C, the surface-adsorbed water was not fully desorbed and affected the interaction with NO. The band at 3226 cm^−1^ is due to hydrogen-bonded OH groups. The negative bands developed during the gas exposure time at 1630 cm^−1^ and in the range of 3580–3700 cm^−1^. The band at 1630 cm^−1^ is due to bending H_2_O vibration modes and the sharp bands between 3580 and 3700 cm^−1^ can be attributed to non-hydrogen-bonded hydroxyl groups and indicated as ‘‘free’’ OH groups on the surface [63,64,65,66,67,68,69,70]. These negative band changes are due to the interaction and/or replacement of hydroxyls with adsorbed NO_x_^−^ species. As mentioned in literature, the formation of NO_x_^−^ anions is often accompanied by releasing of water because the NO_x_^−^ species replace surface OH groups [64,65,66]. Also, NO_x_^−^ species are produced on wet surfaces and previously adsorbed water leads to a shift the frequency of N-O stretching modes toward lower wavenumbers [67,68]. A summary of the absorption bands upon NO exposure is presented in Table 3.

Comparison of DRIFT spectra for unmodified semiconductor oxides and hybrids reveals the role of RuITP complex in the interaction of sensitive materials with NO. Compared to SnO_2_ spectrum, DRIFT spectrum of SnO_2_+RuITP is characterized by increased intensity of oscillation bands with frequencies of 1540 cm^−1^ and 1592 cm^−1^ corresponding to monodentate NO_3_^−^_(ads)_ and chelating bidentate NO_3_^−^_(ads)_ respectively. The growth of NO_3_^−^_(ads)_ concentration is determined by Equations (5) and (6). Equation (5) involves the interaction of the sensitive material with NO_2(gas)_. Therefore, an increase in the concentration of NO_3_^−^_(ads)_ may indicate an increase in the NO_2_ concentration in the NO + air gas mixture due to reaction

2NO_(gas)_ + O_2(gas)_ = NO_2(gas)_. (11)

It is known that ruthenium compounds are active catalysts for the oxidation of nitrogen compounds [71,72,73,74]. This catalytic effect was also noted in the detection of nitrogen containing gases by SnO_2_ based gas sensitive materials [75,76]. At the same time, the sensitivity of *n*-type semiconductor oxides to NO_2_ significantly exceeds their sensitivity to NO [23]. Thus, an increase in the resistive response of SnO_2_+RuITP (compared to unmodified SnO_2_) towards NO may be due to the catalytic action of RuITP in the oxidation of NO to NO_2_ in the gas phase.

Contrary, In_2_O_3_+RuITP and In_2_O_3_ demonstrate very close values of the resistive response toward NO. Comparing the DRIFT spectra of these samples, in the case of In_2_O_3_+RuITP, we can note an increase in the intensity of oscillations with a frequency of 1220 cm^−1^, corresponding to bridging bidentate NO_2_^−^ and the appearance of oscillations with a frequency of 1420 cm^−1^, due to the formation of nitro groups NO_2_^−^. This indicates a change in the coordination of some of the chemisorbed NO_2_^−^_(ads)_ species. In this case, there is no change in the intensity of oscillations corresponding to NO_3_^−^_(ads)_. Thus, in In_2_O_3_ based hybrid material the catalytic action of RuITP in the oxidation of NO to NO_2_ in the gas phase is not shown.

### 3.7. Effective Photoresponse

Unmodified SnO_2_ does not exhibit photosensitivity under blue light illumination and shows very low change in photoresponse with the increase in NO concentration (Figure 10e). At the same time unmodified In_2_O_3_ exhibits high photoresponse, which strongly depends on NO concentration (Figure 10f). The photosensitivity of In_2_O_3_ may be due to the excitation of electrons from acceptor levels lying in the forbidden band of metal oxide. Under visible light illumination electrons are excited from such levels into the conduction band of the semiconductor, the thermal equilibrium in the system is violated, as a result of which, the electrons must be excited from the valence band into acceptor levels in order to recover this equilibrium. The result of this process will be the formation of holes in the valence band of the semiconductor, which can cause photodesorption of gas molecules from the surface.

**Figure 13:**
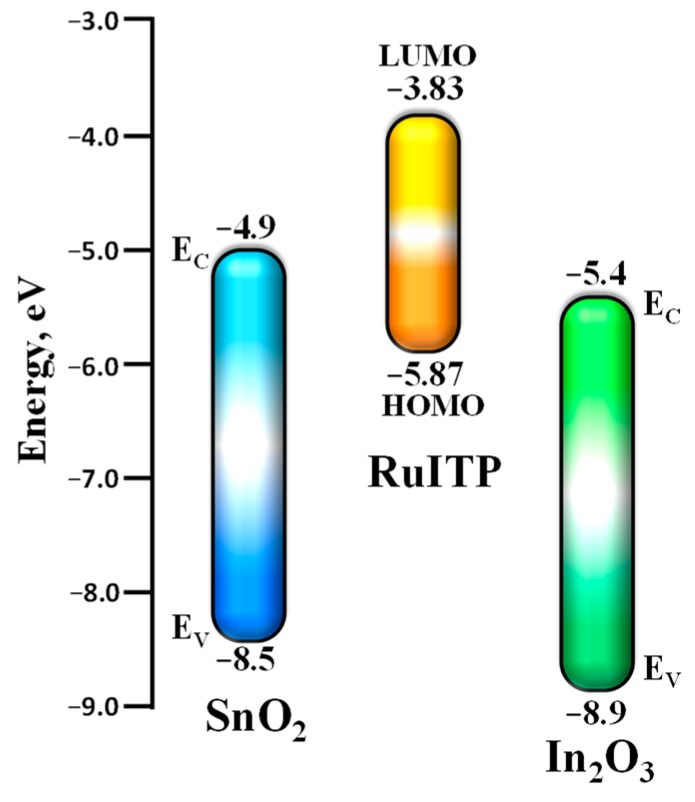
Scheme of the mutual arrangement of the energy levels for bulk SnO_2_, In_2_O_3_, HOMO, and LUMO of the RuITP complex.

Surface modification of SnO_2_ and In_2_O_3_ with RuITP complex leads to the growth of photosensitivity accompanied by the reproducible change in photoresponse depending on NO concentration (Figure 10e,f). The ratios of effective photoresponse *S_ph_* in the presence of 4 ppm NO for hybrid materials and corresponding unmodified oxides are *S_ph_*(SnO_2_+RuITP)/*S_ph_*(SnO_2_) = 2.2 and *S_ph_*(In_2_O_3_+RuITP)/*S_ph_*(In_2_O_3_) = 5.5. The greater amplification of the photoresponse in the case of In_2_O_3_ sensibilization is due to the mutual arrangement of the energy levels for bulk SnO_2_, In_2_O_3_, HOMO, and LUMO of the RuITP complex (Figure 13.) The positions of the respective valence band (*E_V_*) and conduction band (*E_C_*) were taken from the literature [25,77,78,79]. In this case, photoexcited charge carriers, which are generated upon irradiation of hybrids with the light corresponding to the absorption band of Ru (II) heterocyclic complex, play a key role in ensuring the reversible interaction of the hybrids with the gas phase. During photoexcitation of Ru (II) heterocyclic complex, electron-hole pairs are generated in them and electrons are transferred from the LUMO level of the RuITP complex to the conduction band of the oxide matrix, which is lower in energy. As a result, the conductivity will increase. The bottom of the In_2_O_3_ conduction band lies about 0.5 eV below the bottom of the SnO_2_ conduction band. This leads to an increase in the energy difference between *E_С_* In_2_O_3_ and LUMO of the RuITP complex from which the electron is injected (*∆E* = LUMO—*E_С_* is 1.1 eV for SnO_2_ and 1.6 eV for In_2_O_3_). According to Marcus theory of electron transfer [80], the larger *∆E* value will correspond to the larger rate constant of the electron transfer into the semiconductor matrix. This explains the more efficient sensitization of In_2_O_3_ matrix compared to SnO_2_.

At the same time, photoexcited holes remain in the HOMO level of the organic complex, which undergo recombination with electrons localized in chemisorbed species of oxidizing gases. These species lose their charge and become weakly bonded. As a result, photodesorption of oxidizing gases under visible light becomes possible (Equation (8) and (10)).

**Figure 14:**
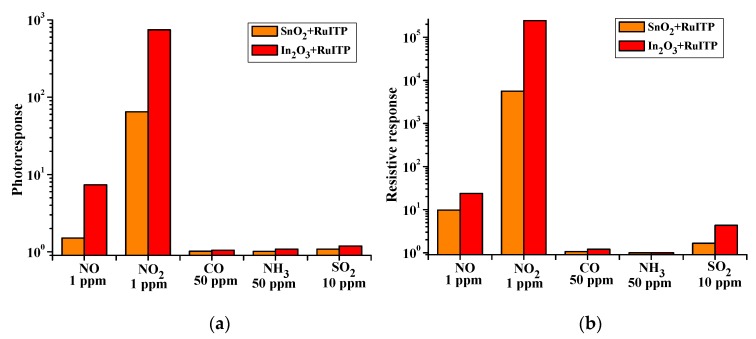
Comparison of the effective photoresponse (**a**) and resistive response (**b**) of hybrid materials when detecting different gases at room temperature under periodic blue light illumination.

Figure 14 shows a comparison of the effective photoresponse (a) and resistive response (b) of hybrid materials when detecting different gases at room temperature under periodic blue light illumination. As follows from the DRIFT spectra discussed above and the literature data [23], the interaction of NO and NO_2_ with the surface of semiconductor oxides occurs in a similar way, through the mechanism of adsorption with the localization of electrons of the semiconductor conduction band. However, the lower sensitivity of hybrid materials to NO, compared to NO_2_, should be due to different initial steps in the detection routes. In reaction with NO_2_, it is a simple one-electron reduction (Equation (4)) favored by the strong oxidative activity of nitrogen dioxide. The interaction with NO (Equation (7)) is essentially an oxidation of the target gas mediated by oxygen on the surface of the semiconductor oxide. This should be the main reason for the different sensitivities to NO_2_ and NO, although the surface species produced in both interaction pathways are similar [23].

The hybrid materials show very low sensitivity to CO and NH_3_ (Figure 14). As it was mentioned in the Introduction, for the detection of these reducing gases, the presence of chemisorbed oxygen on the surface of the semiconductor oxide is necessary. At the same time, the use of illumination leads to photodesorption of oxygen (Equation (9)) and, consequently, a decrease in the sensitivity of materials to CO and NH_3_.

An interesting fact is the noticeable sensor signal of hybrid materials to SO_2_. This may be due to the fact that on the surface of semiconductor oxides, SO_2_ and NO_2_ adsorb in a similar way, and the surface sulfate groups formed during SO_2_ adsorption can be desorbed only at high temperatures [81,82]. The higher basicity of In_2_O_3_ compared to SnO_2_ provides a higher sensor response of the In_2_O_3_+RuITP hybrid material to the acid gas SO_2_.

Thus, at room temperature under periodic blue light illumination, hybrid materials SnO_2_+RuITP and In_2_O_3_+RuITP will be able to detect NO (in sub-ppm concentration range) in the presence of high concentration (tens of ppm) of a wide range of reducing gases, the detection of which is due to their oxidation by chemisorbed oxygen on the surface of semiconductor oxides. At the same time, in the presence of such strong electronic acceptors as NO_2_ and O_3_ [23,27,83], selective detection of NO will be impossible. However, neither NO_2_ nor ozone are found in the exhaled air [84]. Thus, hybrid materials SnO_2_+RuITP and In_2_O_3_+RuITP are extremely promising for the analysis of NO concentration in exhaled air for noninvasive diagnosis of respiratory diseases.

The stability of the sensors was tested under the conditions of periodic measurements for 2 months (Figure S8, Supplementary Materials). Before each test, the sensors were kept in dry air for 2 h under periodic illumination. The values of the photoresponse and resistive response of hybrid materials to a fixed NO concentration vary within 10 rel.%. For unmodified matrices SnO_2_ and In_2_O_3_, a gradual decrease in the values of the photoresponse and the resistive response was found (on average by 30 rel.% in 60 days), as well as an increase in the resistance of these materials in pure air. This may be due to incomplete desorption of NO_x_^−^ species from the surface of semiconductor oxides under blue light.

So, comparing the values of the sensor response (Figure 10), selectivity (Figure 14), and stability (Figure S8, Supplementary Materials) of the studied materials, we can conclude that the hybrid material In_2_O_3_+RuITP is the most promising for practical application.

## 4. Conclusions

Synthesized organo-inorganic hybrid materials based on nanocrystalline oxides SnO_2_ and In_2_O_3_ and heterocyclic Ru (II) complex (RuITP) showed high sensitivity in detecting nitrogen monoxide NO (0.25–4.0 ppm) at room temperature under periodic illumination with blue light (λ_max_ = 470 nm). The resistive response calculated as the ratio *S* = *R_dark gas_*/*R_dark air_* of dark resistances and effective photoresponse *S_ph_* = *R_dark_*/*R_light_* were used as the sensor signal.

Modification of semiconductor oxides by the RuITP complex differently affects the sensitivity of SnO_2_ and In_2_O_3_ to NO under dark conditions. The LDL_NO_ values calculated from resistive response for SnO_2_+RuITP and In_2_O_3_+RuITP samples were 69 ppb and 88 ppb, respectively. Based on the results of in situ DRIFT analysis it was assumed, that an increase in the resistive response of SnO_2_+RuITP (compared to unmodified SnO_2_) towards NO may be due to the catalytic action of RuITP in the oxidation of NO to NO_2_ in the gas phase.

Surface modification of SnO_2_ and In_2_O_3_ with RuITP complex leads to the growth of photosensitivity accompanied by the reproducible change in photoresponse depending on NO concentration. The ratios of *S_ph_* in the presence of 4 ppm NO for hybrid materials and corresponding unmodified oxides are *S_ph_*(SnO_2_+RuITP)/*S_ph_*(SnO_2_) = 2.2 and *S_ph_*(In_2_O_3_+RuITP)/*S_ph_*(In_2_O_3_) = 5.5. The greater amplification of the photoresponse in the case of In_2_O_3_ sensibilization is explained by the larger *∆E* = LUMO(RuITP)—*E_С_*(In_2_O_3_) value providing larger rate constant of the photoelectron transfer from Ru (II) complex into semiconductor matrix.

## Figures and Tables

**Table 1:** Microstructure characteristics of synthesized semiconductor oxides.

Sample	Phase Composition	d_XRD_^(a)^, nm	d_TEM_^(b)^, nm	S_surf_^(c)^, m^2^/g	Average Pore Diameter, nm
SnO_2_	SnO_2_, cassiterite	4 ± 1	4 ± 1	115 ± 5	3–5; 70–80
In_2_O_3_	In_2_O_3_, bixbyite	7 ± 1	7 ± 2	90 ± 5	3–4

^(a)^ crystallite size from XRD; ^(b)^ particle size from TEM; ^(c)^ specific surface area.

**Table 2:** Assignment of Raman vibrational modes (cm^−1^).

SnO_2_	In_2_O_3_	SnO_2_+RuITP	In_2_O_3_+RuITP
137 (B_1g_)	132 (A_g_)	141 (B_1g_)	307.8 (T_g_)
309, 352 (IR E_u_)	307.7 (T_g_)	245.7 (IR)	433 (surface mode)
444 (IR B_1u_)	366.7 (T_g_)	299, 344.7 (IR E_u_)	484 (A_g_)
480.5 (E_g_)	458 (surface mode)	497.5 (E_g_)	665.6 (T_g_)
563 (surface mode)	494.6 (A_g_)	543.5 (surface mode)	930 (ring breathing)
631 (A_1g_)	628.5 (T_g_)	620 (A_1g_)	1028.6 (δ(ring) + ν(Ru-N))
772.8 (B_2g_)		681 (Ru-N str., ring breathing)	1071.4, 1098.5 (δ(CH) + ν(ring))
		755.6 (B_2g_), ring breathing)	1187 (δ(CH))
		1024 (δ(ring) + ν(Ru-N))	1218, 1255 (ν(C=N) + ν(C=C) + δ(CH))
		1168, 1186.5 (δ(CH))	1311.5, 1345 (ν(C-C) + δ(CH))
		1265 (ν(C=N) + ν(C=C) + δ(CH))	1434 (δ(CH) + ν(C=N) chel.)
		1314, 1364 (ν(C-C) + δ(CH))	1479 (ν(C-C), in phase)
		1433.6 (δ(CH) + ν(C=N) chel.)	1540, 1558 (ν(C=C), ν(C=N))
		1482.7 (ν(C-C), in phase)	1600.8 (ν(C=C), ν(C-C))
		1556 (ν(C=C), ν(C=N))	
		1600.6 (ν(C=C), ν(C-C))	

**Table 3:** Assignments of IR bands (cm^−1^) appeared in DRFIT spectra as a result of NO adsorption on the surface of prepared samples at room temperature according to [60,69,70].

Sample	Wavenumber, cm^−1^	Assignments
SnO_2_/SnO_2_+RuITP	1050, 1285, 1540	monodentate NO_3_^−^
	1450	monodentate NO_2_^−^
	1592	chelating bidentate NO_3_^−^
	1630	bending H_2_O
	3600–3700	‘‘free’’ OH groups
In_2_O_3_/In_2_O_3_+RuITP	1036, 1240, 1285	monodentate NO_3_^−^
	1145, 1175	chelating bidentate NO_2_^−^
	1198, 1220	bridging bidentate NO_2_^−^
	1420	nitro compound NO_2_^−^
	1075, 1450, 1475	monodentate NO_2_^−^
	1575	chelating bidentate NO_3_^−^
	1630	bending H_2_O
	3226	H-bonded OH groups
	3580–3700	‘‘free’’ OH groups

## Supplementary Materials

The following are available online at https://www.mdpi.com/2079-4991/10/1/70/s1, Figure S1: Structure of the 5-(1H-imidazo[4,5-f][1,10]phenanthrolin-2-yl)thiophene-2-carbaldehyde and 2,5-bis(1H-imidazo[4,5-f][1,10]phenanthrolin-2-yl)thiophene; Figure S2: Structure of the (E)-4-(2-(5-(1H-imidazo[4,5-f][1,10]phenanthrolin-2-yl)thiophen-2-yl)vinyl)-1-methylpyridin-1-ium iodide; Figure S3: Structure of the bis(2,2′-bipyridin-k2N1,N1′)[4-{(E)-2-[5-(1H-imidazo[4,5-f][1,10]phenanthrolin-2-yl)thiophen-2-yl]ethynyl}-1-methylpyridinium iodide] ruthenium (II) dichloride; Figure S4: Voltammogram of the RuITP complex; Figure S5: XRD patterns of nanocrystalline SnO_2_ (**a**) and In_2_O_3_ (**b**) samples; Figure S6: N_2_ adsorption-desorption isotherms for nanocrystalline In_2_O_3_ and SnO_2_ powders; Figure S7: Pore size distribution (BJH) for nanocrystalline In_2_O_3_ and SnO_2_ powders; Figure S8: Change in photoresponse (**a**) and resistive response (**b**) of nanocrystalline oxides SnO_2_ and In_2_O_3_ and hybrids SnO_2_+RuITP and In_2_O_3_+RuITP in the presence of 4 ppm NO within 60 days; Table S1: Electrochemical data and calculated values of HOMO and LUMO of RuITP complex in MeCN with 0.1 M TBAP as supporting electrolyte; potentials were measured relative to Ag|AgCl|KCl aq. sat. reference electrode.
