# Supplementary material for: Organic-Inorganic Hybrid Materials for Room Temperature Light-Activated Sub-ppm NO Detection
Source: Nanomaterials (Basel). 2019 Dec 28;10(1):70. doi: 10.3390/nano10010070 (PMC7023258; doi:10.3390/nano10010070)
Supplement: Supplementary file 1 [file nanomaterials-10-00070-s001.pdf]

Supplementary Materials: Organic-Inorganic Hybrid Materials for Room Temperature Light-Activated Sub-ppm NO Detection

Abulkosim Nasriddinov [1,2], Marina Rumyantseva [1,*], Tatyana Shatalova [1], Sergey Tokarev [1,3], Polina Yaltseva [1], Olga Fedorova [1,3], Nikolay Khmelevsky [4] and Alexander Gaskov [1]

[1] Chemistry Department, Moscow State University, 119991 Moscow, Russia; naf_1994@mail.ru (A.N.); shatalovatb@gmail.com (T.S.); pergeybokarev@gmail.com (S.T.); yal-polina@yandex.ru (P.Y.); fedorova@ineos.ac.ru (O.F.); gaskov@inorg.chem.msu.ru (A.G.)
[2] Faculty of Materials Science, Moscow State University, 119991 Moscow, Russia
[3] A.N. Nesmeyanov Institute of Organoelement Compounds RAS, 119991 Moscow, Russia
[4] LISM, Moscow State Technological University Stankin, 127055 Moscow, Russia; khmelevsky@mail.ru
* Correspondence: roum@inorg.chem.msu.ru; Tel.: +7-495-939-5471

Synthesis of Ru (II) complex

The synthesis of heteroleptic Ru (II) complex bis(2,2′-bipyridin-k^{2}N^1,N$^{1'}$)[4-{(E)-2-[5-(1H-imidazo[4,5-f][1,10]phenanthrolin-2-yl)thiophen-2-yl]ethynyl}-1-methylpyridinium iodide] ruthenium (II) dichloride. (Ru-ITP) (IX) was prepared by several steps as shown in Schemes S1-S3.

First, 1,4-dimethylpyridinium iodide (I) was obtained by alkylation of 4-methylpyridine with methyl iodide for 3 hours at a temperature of 50 °C as described elsewhere [1] (Scheme S1).

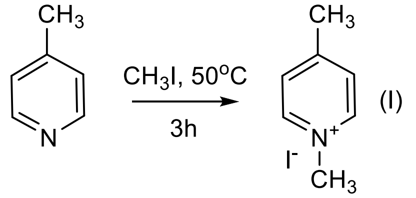

(S1)

Next the 5-(1H-imidazo[4,5-f][1,10]phenanthroline-2-yl)thiophene-2-carbaldehyde (IV) was obtained by condensation of 1,10-phenathroline-5,6-dione (II) and thiophene-2,5-dicarbaldehyde (III) in the presence of ammonium acetate in glacial acetic acid. The technique was based on [2] Product formation is accompanied by the producing of side compound 2,5-di(1H-imidazo[4,5-f][1,10]phenanthrolin-2-yl)thiophene (V) in a 4:1 (IV:V) ratio. The next stage was carried out in a sealed ampoule in ethanol; pyrrolidine was used as the base. Synthesis of (E)-4-(2-(5-(1H-imidazo[4,5-f][1,10]phenanthrolin-2-yl)thiophen-2-yl)vinyl)-1-methylpyridin-1-ium iodide ligand (VII) was carried as shown in Scheme S2:

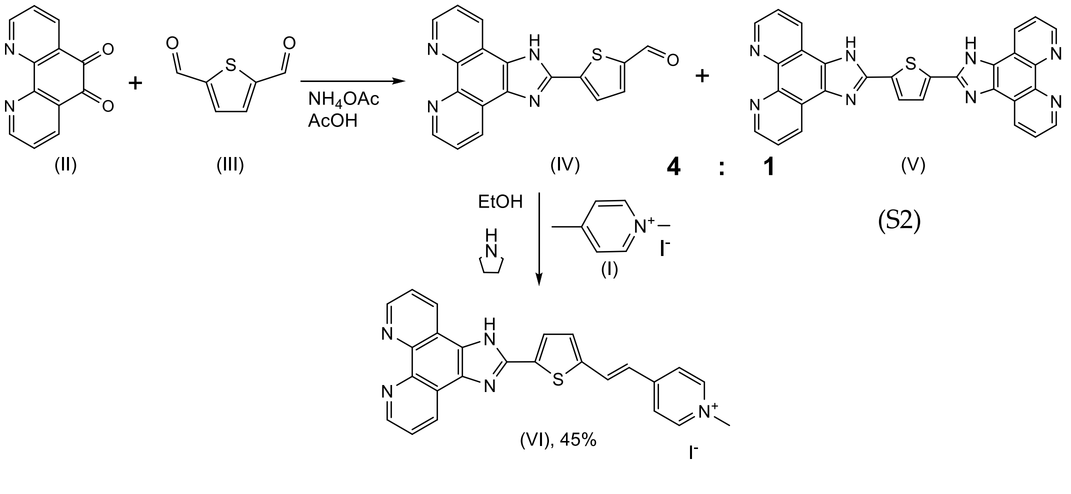

(S2)

In order to obtain the ruthenium complex an equimolar amount of ligand (VII) was kept with cis-bis(2,2-bipyridine)dichlororuthenium II hydrate [3] in ethanol at 80 °C in sealed ampoule in argon for 8 h (Scheme S3). After complete reaction, the column chromatography was used to purify the crude complexes.

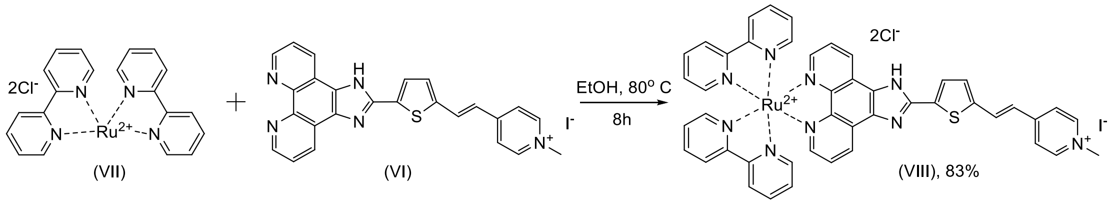

(S3)

The synthesized ligand and its respective heteroleptic ruthenium complex were characterized by ^{1}H NMR, ^{13}C NMR, MALDI-TOF mass spectrometry and elemental analysis.

^{1}H and ^{13}C (APT method) NMR spectra were recorded on a Bruker AVANCE-400 and Avance-500 spectrometers (Bruker, Mundelein, Illinois, USA). The chemical shifts and spin-spin coupling constants were determined with accuracy of 0.01 ppm and 0.1 Hz, respectively. Two-dimensional techniques HMQC, HMBC and COSY were used for the assignment of signals in the spectra.

ESI mass spectra (ESI-MS) were acquired on a Finnigan LCQ Advantage tandem dynamic mass spectrometer (San Diego, CA, USA) equipped with a mass analyzer with an octapole ionic trap, a MS Surveyor pump, a Surveyor autosampler, a Schmidlin-Lab nitrogen generator (SRI Instruments GmbH, Bad Honnef, Germany), and a system of data collection and processing using the X Calibur program, version 1.3 (Finnigan). The mass spectra were measured in the positive ion mode. Samples in MeCN were injected directly into the source at flow rate 50 μL min-1 through a Reodyne injector with a loop of 20 μL. The temperature of the transfer capillary was 150 °C, and the electrospray needle was held at potential 4.0 kV.

Elemental analysis was performed on a Carlo Erba 1108 elemental analyzer (Okehampton, West Devon, UK).

Ligand:

Synthesis of (E)-4-(2-(5-(1H-imidazo[4,5-f][1,10]phenanthrolin-2-yl)thiophen-2-yl)vinyl)-1-methylpyridin-1-ium iodide (VI)

Synthesis of 5-(1H-imidazo[4,5-f][1,10]phenanthrolin-2-yl)thiophene-2-carbaldehyde (IV) and 2,5-bis(1H-imidazo[4,5-f][1,10]phenanthrolin-2-yl)thiophene (V)

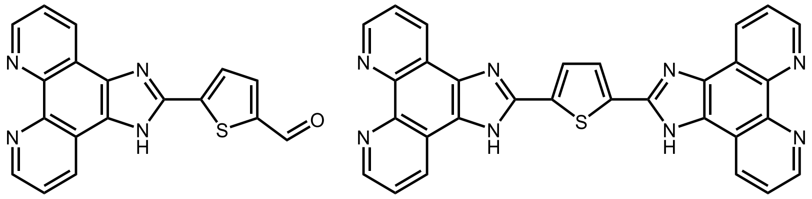

(IV) (V)

Figure S1. Structure of the 5-(1H-imidazo[4,5-f][1,10]phenanthrolin-2-yl)thiophene-2-carbaldehyde and 2,5-bis(1H-imidazo[4,5-f][1,10]phenanthrolin-2-yl)thiophene

1,10-phenanthroline-5,6-dione (2.7 mmol, 560 mg), thiophene-2,5-dicarbaldehyde (3 mmol, 420 mg) and ammonium acetate (0.06 mol, 4.63 g) were dissolved in 8 ml of glacial acetic acid. The mixture was stirred for 6 hours at reflux. After the reaction was completed an aqueous solution of NH_4OH (13%) was added to pH ~ 8. The precipitate formed was filtered on a glass porous filter, washed with water and diethyl ether and dried on a rotary evaporator at 75 °C. The pure mixture of condensation products (IV) and (V) in a ratio 4:1 respectively was obtained as a beige precipitate (760 mg). The mixture without separation was used in the reaction of preparation of ligand (VI). The yield of the desired product is about 60% calculated by 1,10-phenanthroline-5,6-dione.

^{1}H NMR (DMSO-d6, δ; ppm, J/Hz) (IV): 7.64 (dd, 2H, ^{3}J =4.3, ^{3}J =8.1, ImPh), 7.77 (d, 1H, ^{3}J =3.9, Th), 7.95 (d, 1H, ^{3}J =4.0, Th), 8.77 (d, 2H, ^{3}J =8.0, ImPh), 8.83 (d, 2H, ^{3}J =4.4, ImPh), 9.84 (s, 1H, CHO).

^{1}H NMR (DMSO-d6, δ; ppm, J/Hz) (V): 7.79 (m, 4H, ImPh), 7.90 (s, 2H, Th), 8.89 (d, 4H, ^{3}J =8.2, ImPh), 8.96 (d, 4H, ^{3}J =4.7, ImPh).

Synthesis of (E)-4-(2-(5-(1H-imidazo[4,5-f][1,10]phenanthrolin-2-yl)thiophen-2-yl)vinyl)-1-methylpyridin-1-ium iodide (VI)

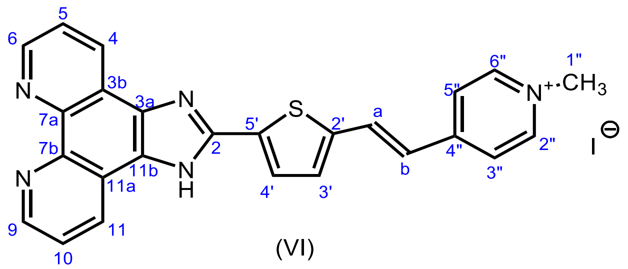

(VI)

Figure S2. Structure of the (E)-4-(2-(5-(1H-imidazo[4,5-f][1,10]phenanthrolin-2-yl)thiophen-2-yl)vinyl)-1-methylpyridin-1-ium iodide.

To a solution of mixture (650 mg) of aldehyde (IV) and binary (V) in 21 ml of absolute ethanol there were added 1,4-dimethylpyridin-1-ium iodide (1.3 mmol, 300 mg) and 0.5 ml of pyrrolidine. The reaction was carried out in a sealed tube for 2 hours at 100 ˚C. The tube was opened and the ethanol was evaporated on a rotary evaporator, the resulting viscous mass was boiled in 15 ml of acetonitrile for 15 min. The insoluble precipitate was separated by filtration, the desired product was washed off the filter with methanol. Methanol was evaporated on a rotary evaporator. 402 mg of the desired product (VI) were obtained as a dark purple powder. The yield is 45%.

^{1}H NMR (DMSO-d6, δ; ppm, J/Hz): 4.17 (s, 3H, CH_3(1″)), 7.11 (d, 1H, ^{3}J=15.9, CH=CH(b)), 7.47 (d, 1H, ^{3}J=3.8, Th(3′)), 7.71 (dd, 2H, ^{3}J=7.8, ^{3}J=4.7, ImPh(5,10)), 7.77 (d, 1H, ^{3}J=3.8, Th(4′)), 8.10 (d, 2H, ^{3}J=6.9, Py(3″,5″)), 8.19 (d, 1H, ^{3}J=15.9, CH=CH(a)), 8.72 (d, 2H, ^{3}J=6.9, Py(2″,6″)), 8.84 (m, 4H, ImPh(6,9), ImPh(4.11)).

^{13}C NMR APT (DMSO-d6, δ; ppm): [CH_3]: 46.55 (CH_3(1″)), [CH]: 120.07 (CH=CH(a)), 122.58 (Py(3″,5″)), 122.75 (ImPh(5,10)), 124.18 (Th(4′)), 129.72 (ImPh(4,11)), 133.87 (Th(3′)), 134.54 (CH=CH(b)), 144.52 (Py(2″,6″)), 145.85 (ImPh(6,9)), [C]: 124.32 (ImPh(3b,11a)), 136.25 (ImPh(3a,11b)), 138.23 (Th(2′)), 142.74 (ImPh(7a,7b)), 145.42 (ImPh(2)), 152.45 (Py(4″)).

MALDI-MS m/z: 420.2 $[M]^+$.

Calculated for $C_{25}H_{18}N_5S$ (%): C, 71.41; H, 4.31; N, 16.65, found (%): C, 71.48; H, 4.28; N, 16.83.

Complex:

Synthesis of bis(2,2′-bipyridin-k2N1,N1′)[4-{(E)-2-[5-(1H-imidazo[4,5-f][1,10]phenanthrolin-2-yl)thiophen-2-yl]ethynyl}-1-methylpyridinium iodide] ruthenium (II) dichloride (VIII)

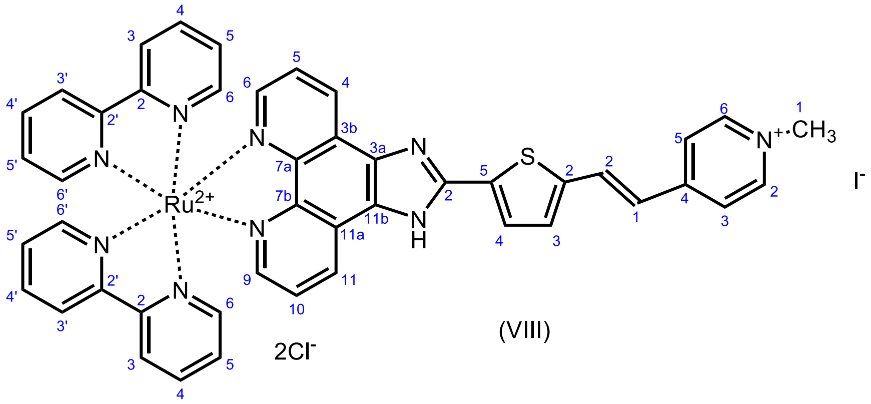

(VIII)

Figure S3. Structure of the bis(2,2′-bipyridin-k2N1,N1′)[4-{(E)-2-[5-(1H-imidazo[4,5-f][1,10]phenanthrolin-2-yl)thiophen-2-yl]ethynyl}-1-methylpyridinium iodide] ruthenium (II) dichloride.

A solution of the (E)-4-(2-(5-(1H-imidazo[4,5-f][1,10]phenanthrolin-2-yl)thiophen-2-yl)vinyl)-1-methylpyridin-1-ium iodide (VI) (0.18 mmol, 100 mg) and *cis*-bis(2,2-bipyridine)dichlororuthenium (II) hydrate (VII) (0.18 mmol, 81 mg) in 10.5 ml of absolute ethanol was placed in a tube, degassed 3 times and filled with argon. The tube was sealed and kept at 80 °C for 8 hours. Next, the tube was opened and

the solvent was evaporated on a rotary evaporator. The resulting crude product was purified by column chromatography on alumina, eluent—a mixture of benzene: methanol in a ratio of 1:1. 145 mg (0.15 mmol) of pure product were obtained with the yield of 83% as a dark violet precipitate.

^{1}H NMR (methanol-d4, δ; ppm, J/Hz): 4.28 (s, 3H, Me), 7.18 (d, 1H, ^{3}J=15.9, CH=CH(1)), 7.33 (t, 2H, ^{3}J=6.1, bpy (5′)), 7.55 (m, 3H, Th (3), bpy(5)), 7.7 (d, 2H, ^{3}J=5.6, bpy(6′)), 7.74 (dd, 2H, ^{3}J=8.4, ^{3}J=5.2, ImPh(5,10)), 7.92 (d, 1H, ^{3}J=4.0, Th (4)), 7.94 (d, 2H, ^{3}J=5.2, ImPh(6,9)), 7.96 (d, 2H, ^{3}J=5.6, bpy(6)), 8.05 (t, 2H, ^{3}J=8.1, ^{3}J=7.9, bpy(4′)), 8.11 (d, 2H, ^{3}J=6.9, bpy(3,5)), 8.17 (m, 3H, CH=CH(2), bpy(4)), 8.65 (d, 2H, ^{3}J=6.9, bpy(2,6)), 8,72 (d, 2H, ^{3}J=8.1, bpy(3′)), 8,76 (d, 2H, ^{3}J=8.1, bpy(3)), 9.13 (d, 2H, ^{3}J=8.2, ImPh(4,11)).

^{13}C NMR APT (methanol-d4, δ; ppm): 46.17 ($\underline{C}H_3$), [CH]: 120.68 (CH=$\underline{C}$H(1)), 123.03 (bpy(3,5)), 124.10 (bpy(3′)), 124.17 (bpy(3)), 124.96 (ImPh(5,10)), 125.98 (Th(4)), 127.38 (bpy(5′)), 127.45 (bpy(5)), 130.34 (ImPh(4,11)), 133.25 ((Th(3)), 134.56 ($\underline{C}$H=CH(2)), 137.54 (bpy(4′)), 137.68 (bpy(4)), 144.41 (bpy(2,6)), 147.62 (ImPh(6,9)), 151.20 ((bpy(6′)), 151.42 (bpy(6)); [C]: 125.85 (ImPh(3b,11a)), 137.86 (ImPh(3a,11b)), 140.19 (Th(2)), 142.95 (ImPh(2)), 144.72 (ImPh(7a,7b)), 153.56 (bpy(4)), 157.23 (bpy(2′)), 157.45 (bpy(2)).

ESI-MS m/z: 417 $[M\text{-}2Cl^-\text{-}I^-\text{-}H^+]^{2+}$.

Calculated for $C_{45}H_{34}Cl_2IN_9RuS$ (%): C, 52.38; H, 3.32; N, 12.22; Ru, 9.80, found (%): C, 52.47 H, 3.38; N, 12.17; Ru, 9.71

The voltammogram of the Ru-ITP complex is shown in **Figure S4**. Electrochemical data from the voltammogram of Ru-ITP complex is presented in **Table S1**. The first cathode peak is irreversible and, apparently, the initial reduction proceeds with the participation of the quaternary nitrogen atom. Among the reduction peaks only the second peak is quasi-reversible. Four irreversible oxidation peaks are present in the anode region. The complex's molecule contains iodide and chloride anions, firstly I^-, then two chloride anions ($2Cl^- - 2e \rightarrow Cl_2$) are initially oxidized, and only after oxidation of the halide anions the anode process proceeds along the donor part of the complex. Subsequent peaks are responsible for the oxidation of the ligand fragment. The oxidation potentials are attributed to the one-electron oxidation of the metal-centered highest-occupied molecular orbital (HOMO) (Table S1).

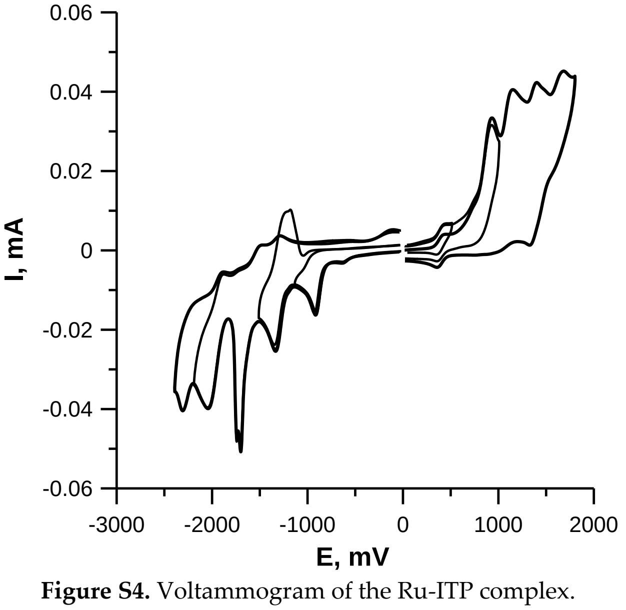

Figure S4. Voltammogram of the Ru-ITP complex.

Table S1. Electrochemical data and calculated values of HOMO and LUMO of Ru-ITP complex in MeCN with 0.1 M TBAP as supporting electrolyte; potentials were measured relative to Ag|AgCl|KCl aq. sat. reference electrode.

$E_{1/2(red)}$, Va	$E_{1/2(ox)}$, Vb	E_{HOMO}, eV	E_{LUMO}, eV
−0.90	0.42		
−1.32/−1.23	0.66 (I^-)		
−1.71 binary	0.92 (Cl^-)	−3.83	−5.87
−2.03	1.14		
−2.30	1.39		
	1.66		

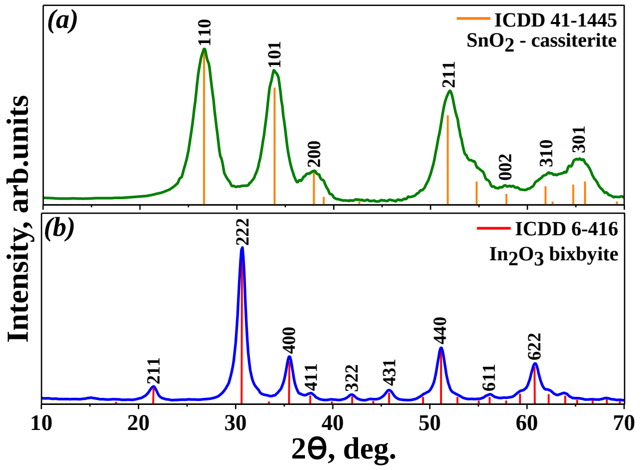

Figure S5. XRD patterns of nanocrystalline SnO_2 (**a**) and In_2O_3 (**b**) samples.

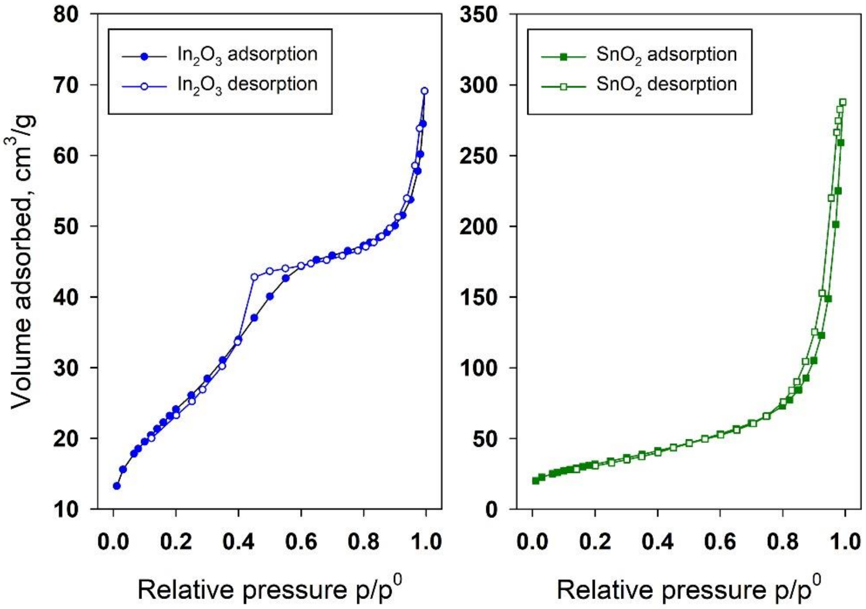

(a) (b)

Figure S6. N_2 adsorption-desorption isotherms for nanocrystalline In_2O_3 (**a**) and SnO_2 (**b**) powders.

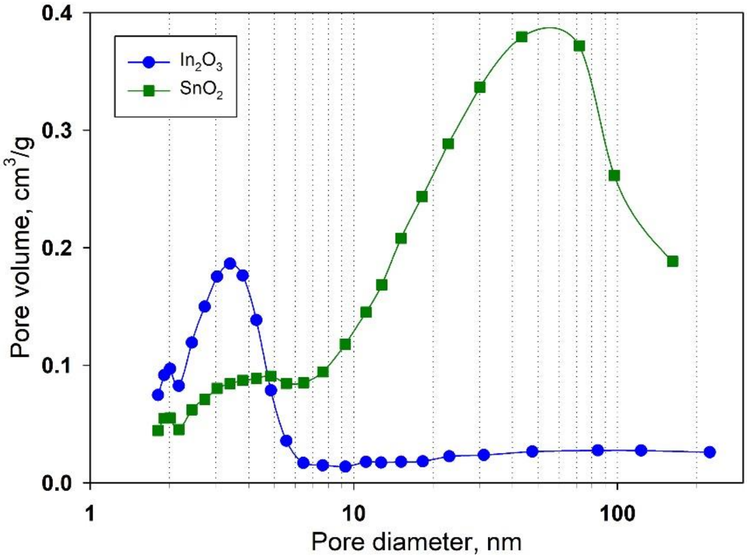

Figure S7. Pore size distribution (BJH) for nanocrystalline In_2O_3 and SnO_2 powders.

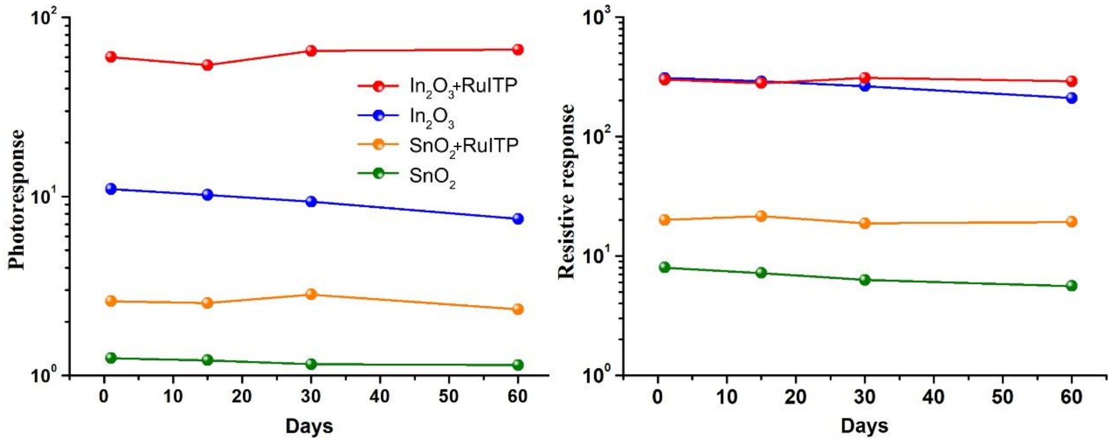

(**a**) (**b**)

Figure S8. Change in photoresponse (**a**) and resistive response (**b**) of nanocrystalline oxides SnO_2 and In_2O_3 and hybrids SnO_2+RuITP and In_2O_3+RuITP in the presence of 4 ppm NO within 60 days.

References

1. Zhang, X.; Li, L.L.; Liu, Y. Fluorescent detection and imaging of Hg^{2+} using a novel phenanthroline derivative based single- and two-photon excitation. *Mater. Sci. Eng. C* **2016**, *59*, 916-923.
2. Day, A.R.; Steck, E.A. Reactions of phenanthraquinone and retenequinone with aldehydes and ammonium acetate in acetic acid solution. *J. Am. Chem. Soc.* **1943**, *65*, 452-456.
3. Wu, J.-Z.; Ye, B.-H.; Wang, L.; Ji L.-N.; Zhou, J.-Y.; Li, R.-H.; Zhou, Z.-Y. Bis(2,2-bipyridine)ruthenium(II) complexes with imidazo[4,5-f][1,10]- phenanthroline or 2-phenylimidazo[4,5-f][1,10]phenanthroline. *J. Chem. Soc., Dalton Trans.* **1997,** 1395-1402
